# Striving for transparent and credible research: practical guidelines for behavioral ecologists

**DOI:** 10.1093/beheco/arx003

**Published:** 2017-03-14

**Authors:** Malika Ihle, Isabel S. Winney, Anna Krystalli, Michael Croucher

**Affiliations:** a Department of Animal and Plant Sciences, Alfred Denny Building, University of Sheffield, Western Bank, Sheffield S10 2TN, UK,; b Max Planck Institute for Ornithology, Eberhard-Gwinner-Strasse, 82319 Seewiesen, Germany,; c Evolution & Diversité Biologique, Bâtiment 4R1, Université de Toulouse Paul Sabatier, 118 Route de Narbonne, 31062 Toulouse Cedex 09, France, and; d Department of Computer Science, Regent Court, University of Sheffield, 211 Portobello, Sheffield S1 4DP, UK

**Keywords:** Acknowledging Open Practices, badges, integrity, open science initiative, software, toolkit, TOP guidelines.

## Abstract

Science is meant to be the systematic and objective study of the world but evidence suggests that scientific practices are sometimes falling short of this expectation. In this invited idea, we argue that any failure to conduct research according to a documented plan (lack of *reliability*) and/or any failure to ensure that reconducting the same project would provide the same finding (lack of *reproducibility*), will result in a low probability of independent studies reaching the same outcome (lack of *replicability*). After outlining the challenges facing behavioral ecology and science more broadly and incorporating advice from international organizations such as the Center for Open Science (COS), we present clear guidelines and tutorials on what we think open practices represent for behavioral ecologists. In addition, we indicate some of the currently most appropriate and freely available tools for adopting these practices. Finally, we suggest that all journals in our field, such as Behavioral Ecology, give additional weight to transparent studies and therefore provide greater incentives to align our scientific practices to our scientific values. Overall, we argue that producing demonstrably credible science is now fully achievable for the benefit of each researcher individually and for our community as a whole.

## Lay Summary

Doubts over the credibility of science can be lifted by open research practices. Low reliability (absence of biases) and reproducibility (transparent workflow) result in a low probability of independent studies reaching the same outcome (lack of replicability). To circumvent these issues, we discuss how the Transparency and Openness Promotion guidelines, proposed by the Center for Open Science, along with a software engineering toolkit allow researchers to embrace the open science process.

Society and researchers themselves seem to be losing confidence in science (Francis 1989; Baker 2016a). Reports of researchers being unable to reproduce results within and between labs and the disproportionate attention that the irreproducible studies receive highlight several problems with how we conduct research (Prinz *et al.* 2011; Begley and Ellis 2012; Open Science Collaboration 2015). Many scientists reading this may wonder “Could my field of research really be unreliable, irreproducible, or non-replicable?” We will first briefly describe how human psychology, a lack of training with new technologies, and a shortage of incentives have affected behavioral ecology, and end with presenting solutions to make our field of research more credible.

*Reliability* is a gold standard in research, and involves researchers objectively addressing a hypothesis. However, as behavioral ecologists, we are particularly aware that animal minds did not evolve to be unbiased in attention, perception, and assessment. Humans, in particular, show widespread evidence of false belief about their abilities (*self-deception*) and selective perception of information that enhances their personal worldview (*confirmation bias*) (Trivers 2011; Lamba and Nityananda 2014). Yet frequently, when managing our research, we ignore our evolutionary predispositions and fail to blind our studies (Holman *et al.* 2015; Kardish *et al.* 2015) or to use systematic and fixed protocols that would ensure our objectivity (John *et al.* 2012; Simmons *et al.* 2011). The term “*researcher degrees of freedom*” encompasses all arbitrary decisions a researcher can take during the course of collecting and analyzing data and embody our most insidious liberty: by refining our studies post-hoc and increasing our number of statistical tests we increase dramatically our probability of a false-positive finding (Simmons *et al.* 2011; Forstmeier *et al.* 2016; Parker *et al.* 2016a). These “questionable research practices” (John *et al.* 2012) (Box 1, a), as opposed to intentional acts of misconduct, offer considerable scope for an individual researcher to rationalize their decisions (Smaldino and McElreath 2016). Therefore, these practices may constitute the vast majority of our research (Parker *et al.* 2016b). Overall, we seem to either fail to conform to scientific standards or tend not to report evidence for our objectivity, making our research outputs unreliable (Leek and Peng 2015).

For a scientific process to be *reproducible*, given the same raw data and same question, someone of equivalent knowledge and using the same methods should be able to reach the same conclusions (Cassey and Blackburn 2006; Patil *et al.* 2016). Given that crowdsourced analyses can produce variable results using the same data and question, being explicit about our methods is essential (Silberzahn *et al.* 2015). Unfortunately, we are generally unable to check the validity of published outcomes because our *workflow* (data extraction, selection, manipulation, analysis, and reporting) is often not disclosed. Imperfect record keeping and nontransparent data processing, as well as lack of portability (i.e., inability to use the same code on different computers), automatization, and appropriate documentation, are undeniably a hindrance to our productivity (Markowetz 2015) (Box 1, b) and can lead to major retractions (Hall and Salipante 2007; Schweppe *et al.* 2008; Pryke *et al.* 2014; Freedman *et al.* 2015; Neimark 2015).

Ultimately, to assess the validity of a finding, close *replications* of published papers are needed (Kelly 2006; Nakagawa and Parker 2015). These studies, closely duplicating a previous one (same population, species, environment, methods), are then used similarly to within-experiment replicates: to better evaluate whether results are due to a true effect, confounding factors, biases, or chance. Once a result is validated, conceptual replications become useful for assessing how general, or repeatable, this finding is across contexts (e.g., testing another prediction of the same hypothesis or testing the same prediction in another species) (Nakagawa and Parker 2015). Unfortunately, in both cases, we may invest substantial effort in replicating and building on a previous study only to realize the absence of an effect (Seguin and Forstmeier 2012; Bulla *et al.* 2015) or to risk being a victim of our aforementioned confirmation bias through “researcher degrees of freedom” (Parker 2013) (Box 1, c). In other words, the current lack of evidence to attest the *reliability* and *reproducibility* of our studies leaves us unable to appropriately assess the likelihood that previous findings are true. In fields such as medicine, neuroscience, and psychology, this has led to a major replication crisis (Freedman *et al.* 2015; Leek and Peng 2015; Open Science Collaboration 2015) and subsequently, to several initiatives to incentivize the validation of important findings (Open Science Collaboration 2012, Reproducibility initiative: http://validation.scienceexchange.com, reproducibility project: https://osf.io/ezcuj/). Replications are often difficult to achieve and can even be misconstrued as potentially damaging to the reputation of the scientist who produced the original results (Ebersole *et al.* 2016). Our field of research currently lacks incentives to promote replication, although many suggestions to promote replication have been proposed and could easily be applied (e.g., “replication reports” and similar [Bruna 2014; Parker and Nakagawa 2014; Endler 2015; Open Science Collaboration, forthcoming; Nakagawa and Parker 2015]). Therefore, replications that test and verify our results have remained rare or nonexistent (Kelly 2006).

Lack of *reliability* and *reproducibility*, combined with the current publication bias against null results, is likely to have generated an over-representation of false-positive evidence (Simmons *et al.* 1999, Ferguson and Heene 2012; Ioannidis 2014; Parker *et al.* 2016a). However, by making our work more reliable and reproducible, we can optimize our *replicability* and prevent the broader replication crisis from submerging our field. Recently, several initiatives have been launched to improve research practices, reduce the false-positive rate to classically assumed levels and once again lend credibility to science (Open Science Collaboration, forthcoming). We focus on these “preventative measures” (Leek and Peng 2015) below.

**Box 1. Is your work affected?**

a) Could you improve your *reliability?* Have you ever:

☐ Neglected to scramble sample identities (or make treatment conditions unidentifiable) before conducting observations, or failed to ask an experienced person who is unaware of the hypothesis to collect the data? (Kardish *et al.* 2015)
☐ Continued sampling after finding a null result because you thought you were lacking the power to detect the expected effect, and did not report this post hoc decision in the final publication? (Simmons *et al.* 2011; Forstmeier *et al.* 2016; Parker *et al.* 2016a)
☐ Reformulated your hypotheses based on what you found and reported this unexpected finding as having been predicted from the start? (Hypothesizing After the Results are Known, or HARKing [Kerr 1998])
☐ Reported only specific dependent measures for a publication and not all the ones you tested? (Simmons *et al.* 2012)
☐ Made a decision to exclude outliers based on the significance of your results before and after exclusion? (Simmons *et al.* 2011)
☐ Tested excluding, including, or transforming covariates with the best intent to describe your data but only presented the final model in your publication? (Simmons *et al.* 2011)

b) Could you benefit from improving your *reproducibility*? Have you ever:

☐ Spent time reprocessing and reanalyzing data without being able to prove (to yourself or someone else) whether all the steps of data processing were identical to the time before?
☐ Found a mistake in your results without knowing where it came from?
☐ Lost parts of datasets or notes on how to process them to obtain the variables of interest?
☐ Forgot what analyses you have already done?

Did you struggle with any of the previous when:

☐ Answering referees’ comments?
☐ New data became available?
☐ Building new projects based on your previous work?
☐ Passing on a project to a team member?
☐ Opening up your project to a collaborator?

c) Could you benefit from *replicable* work? Have you ever:

☐ Based an entire project on a previous interesting finding without first being able to assess its validity?
☐ Been unable to replicate a previously published study (closely or conceptually)?
☐ Been unable to prove that your work or someone else’s was not subject to confirmation bias?

## OPEN SCIENCE: ACCESSIBLE, TRANSPARENT, AND CREDIBLE

The Open Science movement stems from a desire to conduct freely available, reproducible, and reliable science that results in fewer erroneous studies than is currently the case, more certainty in the studies that are published, and greater impact of all our research outputs on science and society. The foundations of the open culture are to conduct research in a clearly defined framework (see Table 1), which allows us to detect and minimize our own biases and blind spots by prompting us with checklists (see for instance the Tools for Transparency in Ecology and Evolution [TTEE], https://osf.io/g65cb/), and promoting working in a single online environment that can be easily shared upon submission, publication, or request (Open Science Collaboration, forthcoming). These initiatives and protocols proposed by the open science community (see below) allow us to test our ideas more objectively, so that we can focus solely on our research and creativity.

**Table 1:** Recommended research process for the main study types in behavioral ecology, with the “minimum” open practices advisable for earning credibility

Study type	Timeline					
	Conception	Collection	Publication	Other research outputs	Cited for	Follow-up studies
Experiment 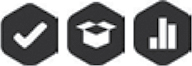	Preregister	Maintain reproducible workflow	Separate confirmatory from exploratory analyses	Open raw data	Objective assessment of an hypothesis	Meta-analysis to quantify the generality of the finding
				Open script for data processing		
			State and follow the 21 word solution^a^	Open script for analyses		
Observational short or middle term 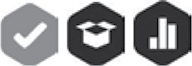	Write project proposal following TOP guidelines and preregistration checklists	Maintain reproducible workflow	State exploratory nature of the analysis	Open raw data Open script for data processing Open script for analyses	Novelty Discovery Hypothesis source	Preregistered replication to test the hypothesis generated 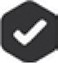
			Briefly report entire exploration			
			State and follow the 21 word solution^a^			
Observational long term 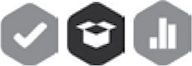	Write project proposal following TOP guidelines and preregistration checklists	Maintain reproducible workflow	State exploratory nature of the analysis	Single study: Open selected raw data	Large sample size Wild population: relevant ecological and evolutionary context	Large-scale collaborations for high impact research
			Briefly report entire exploration State and follow the 21 word solution^a^	Open script for data processing after selection		
				Open script for analyses		
				Full database: Open metadata (prompting preregistrations and subsequent data requests) 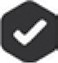		

All these aspects of project management can be carried out and centralized in the Open Science Framework. We highlight some reasons why each study is or would be valuable and commonly cited (either as a result of or without open practices). Further, we emphasize follow-ups that are facilitated or improved by open practices and that promote the impact of the initial research. The open practices are symbolized by “Badges to Akowledge Open Practices” developed by the Center for Open Science and acknowledging Preregistration, Open materials, and Open data, repectively (https://osf.io/tvyxz/wiki/home/). We call journal editors to display them on publications. Preregistration is advisable for all types of studies but alternatives are presented for observational studies where this might be either premature (i.e., when the study is exploratory) or more difficult (e.g., when the data have already been collected and screened by the data analyst). Greyed out badges represent these alternatives and/or cases where an open practice is still lacking incentives in Behavioral Ecology.

^a^The 21 word solution: “We report how we determined our sample size, all data exclusions (if any), all manipulations, and all measures in the study.” (Simmons *et al.*, 2012).

The Center for Open Science (COS; https://cos.io/) is a nonprofit organization dedicated to aligning scientific values and scientific practices (Open Science Collaboration, forthcoming), by encouraging researchers, editors and funding bodies to adopt guidelines promoting transparency and openness (TOP guidelines, https://cos.io/top/, Nosek *et al.* 2015) such as preregistrations (https://cos.io/prereg/), registered reports (https://osf.io/8mpji/), Badges to Acknowledge Open Practices (https://cos.io/rr/), and replications (https://osf.io/wfc6u/). The Open Science Framework (OSF) is an online platform designed by the COS that centralizes all the open practices mentioned above and detailed below and makes the process of managing our research projects easier, by providing us with free, long term, public or private repositories with permanent Digital Object Identifiers (DOI) for datasets and codes (http://help.osf.io/m/60347/l/608740-sharing-data), free training/webinars to teach reproducible tools (https://cos.io/stats_consulting/), and more (https://osf.io/). The OSF integrates multiple open source software packages that have been developed to ensure reproducibility and facilitate collaborations, including Google Drive, GitHub, Dropbox, Figshare, Mendeley, and more (http://help.osf.io/m/gettingstarted).

### Preregistration: essential documentation recommended for all researchers

Preregistration allows us to impartially distinguish exploratory (hypothesis generating) from confirmatory (hypothesis testing) analyses. By registering our detailed study plan (outlining data acquisition, subject exclusion criteria, and preplanned analyses), we can focus on solving our research problems creatively while protecting ourselves from our own “degrees of freedom” that greatly inflate our chances of finding a false-positive result (Open Science Collaboration, forthcoming; Gorgolewski and Poldrack 2016). The COS provides checklists for additional eventualities that we might not (yet) be considering when writing our ordinary project proposal. A date and time stamp is then given to the preregistration, allowing a researcher to prove that tests were conceived a priori, that is, before conducting the study. This initial preregistration can be embargoed for up to 4 years. In specific cases, such as the development of novel methods to address the research question, preregistrations can be updated and receive the label “transparent changes” (TC, https://osf.io/tvyxz/). Any such change can then be seen in the context of the original hypotheses and justified accordingly. In the published paper, any further analyses will be labeled as exploratory and distinguished from the preregistered tests to mark the fact that the investigation has been inspired following analysis of the data. Incidentally, $1000 will be granted to the lead author of the first 1000 preregistered studies published before the end of 2018 (https://cos.io/prereg/#theChallenge). Finally, all preregistrations, even those that the authors wish to withdraw, will be followed up, thus converting unpublished studies into useful scientific resources. Journal editors from all fields, including our own, are being encouraged by the COS to award Badges to Acknowledge Open Practices (Table 1) (Nosek *et al.* 2015). These badges acknowledge the value of preregistered studies and are proof of our *reliability*. By joining other journals in rewarding preregistration (Eich 2014), editors would also contribute substantially to reducing the detrimental practices of HARKing (Hypothesizing After the Results are Known, Box 1, Kerr 1998) and publication bias against “null results” (Forstmeier *et al.* 2016).

Here, we argue that all experiments should be preregistered because these types of studies are, by their very nature, testing hypotheses and contain therefore at least 1 confirmatory analysis (Table 1). This includes long-term experiments that have already started, for which the future series of replications can be preregistered. Furthermore, as the same data cannot be used to both generate and test a hypothesis in a circular fashion, observational studies that put forward hypotheses based on exploratory analyses should be followed by replicative studies such as preregistered experiments or observational studies highlighting one or several preregistered correlations (Table 1). As such, preregistrations are essential for evaluating discoveries made through exploratory analyses. Such replications are the first true tests of the hypothesis at stake. Consequently, we suggest that editors of Behavioral Ecology journals give provisional acceptance to these studies by allowing submission of *registered reports*: studies that are peer reviewed prior to collecting data and observing the study outcome. In addition to the immeasurable benefit of early-stage feedback from our peers, registered reports would considerably reduce publication bias. Finally, we suggest to researchers collecting long-term datasets (for which incentives for open data can appear too weak (Mills *et al.* 2015; Roche *et al.* 2015) that they make preregistration a prerequisite before sharing their data set. Encouraging such requests would promote collaborations and could be achieved by making metadata (data about collected variables) fully available online and without restriction (Table 1). Further comprehensive documentation that advises on study-specific details (such as cohort selection, unusual data points, and more), should also be ready to share. Such high-quality metadata combined with preregistrations would greatly enhance the reproducibility and productivity of long-term studies. Arguably, along with better regulation of data citations, these processes pave the way for opening the raw data themselves by ensuring that the original data set is recognized, finally allowing science to become truly open (Hampton *et al.* 2015). Ultimately, this would benefit the entire scientific community (and beyond) (Evans and Reimer 2009; Boulton *et al.* 2011; Hampton *et al.* 2015; McKiernan *et al.* 2016), as intended by all Open Science organizations (https://science.mozilla.org/, https://ropensci.org/, https://cos.io/).

### Reporting and opening your entire workflow

Another solution to show that our work is reliable and currently the only way of making our work reproducible is to report our entire workflow. We cannot stress enough the importance, for all study types, of fully disclosing “how samples sizes were determined, how many observations, if any, were excluded, all experimental conditions that were tested, including failed manipulations, and all measurements that were taken” (Simmons et al. 2011, 2012; Eich 2014). Beyond these brief statements within the publication, we strongly encourage authors to provide the raw data that support their study during the review process (Baker 2016b), as well as the code needed to process and analyze these data (these are then “truly” open data [Roche *et al.* 2015; Mislan *et al.* 2016]; Table 1). Authors could then receive more insightful and constructive feedback from reviewers. Sharing code, even suboptimally documented code, makes community bug fixes possible, and engages other scientists with the author’s research (Barnes 2010; Gorgolewski and Poldrack 2016). In addition, publications with open data have been shown to receive extra media attention and citations (McKiernan *et al.* 2016). Moreover, these additional research outputs are themselves citable (https://guides.github.com/activities/citable-code/, https://zenodo.org/features, Piwowar 2013) and are therefore legitimate scientific products in their own right (Mislan *et al.* 2016). Overall, open code and open scientific practices lead to research outputs that are inherently easy to share, and therefore promote collaboration.

This entire process can be facilitated and encouraged by journal editors, who can award Badges of Open Data and Open Material (Table 1) in recognition of reproducibility. These Badges have been proven to incentivize scientists to make their work more transparent (Kidwell *et al.* 2016). Editors and reviewers can embrace the open science rigor by requesting complete workflows, code, and data upon submission, as an integral part of a reproducible analysis (Morey *et al.* 2016). Simultaneously, editors and reviewers must keep an open mind and allow clarification and corrections by the authors. Finally, we suggest that journal editors request a statement about whether the study was blinded or not to be included in publications.

Reporting your entire workflow might sound like an impossible task, especially in ecology where large heterogeneous data sets are sometimes combined (Reichman *et al.* 2011). However, most research outputs can, in principle, be fully open upon publication without much effort using the simple and efficient tools presented in the next section.

### Optimize your reproducibility

*“Software is the most prevalent of all the instruments used in modern science”* (Goble 2014) and yet much of our computer programs are developed and used by researchers who have little understanding of even the basics of modern software development (Hannay *et al.* 2009), with many honourable exceptions. This is clearly to our detriment and is easily remedied.

Automation, version control, literate programming, and openness are among the most important software engineering concepts that all scientists should adopt as part of their standard toolkit. The aim of this toolkit is to treat our digital work in the same way as our ideal laboratory: tidy, with well-labeled materials, and appropriate documentation of procedures. Current technologies that implement these concepts include R, RStudio, Git, GitHub and Markdown (Box 2). A tutorial on how to get started with these technologies was developed for the recent post-ISBE 2016 conference symposium “Challenge for our generation: open, reproducible and reliable science” and is available at https://zenodo.org/record/61435#.V9buDK1SVj8.

Those requiring more advanced support can turn toward members of the emerging Research Software Engineering profession (Hettrick 2016), an activity that is now endorsed and financially supported by several research funding bodies (https://www.epsrc.ac.uk/funding/calls/rsefellowships/, Software Sustainability Institute, 2015). Many of the practices developed by software engineers can be directly applied to research data analysis and simulation workflows to improve reproducibility (Ram 2013), correctness (Hampton *et al.* 2015) and accelerate the scientific endeavor (Wilson *et al.* 2014; Wilson *et al.* 2016). The essentials of these initially daunting technologies can be learned in just a few hours or days, as demonstrated by the international Software Carpentry Foundation (Wilson 2014).

**Box 2. One ready-made software engineering toolkit for a behavioral ecologist**

Tool 1: R studio projects. These allow you to:

✓ Have the working directory automatically set to the relevant project folder containing the files for an analysis, which becomes an easily portable directory
✓ Update outputs automatically when any data, data selection rules, or data analyses change
✓ Activate Git version control systems so that the history of the analysis is documented and completely recoverable (see below)
✓ Activate package version control systems such as Packrat to automatically use the packages versions employed at the time of the project, for compatibility with anyone inheriting the project folder

Tool 2: Version control systems like Git. These allow you to:

✓ Keep one unique copy of each code, with an annotated trace of all previous modifications that the file went through (i.e., no need for a version named copy_of_Finalcode_REALFinal_JF20160802_tryloop_brokeloop.R)
✓ Prevent you from ever sending the wrong version of a file, because there is only one
✓ Restore deleted pieces of code, or entire scripts, judged to be suboptimal at the time, when you realize they were not that pointless after all

Tool 3: Platforms for online repositories, like GitHub. These allow you to:

✓ Backup your files every day
✓ Work easily on different computers
✓ Code collaboratively while keeping track of all changes as well as their author
✓ Test new ideas for code without breaking the current one
✓ Receive suggestions for improvement of your own code (potentially also during peer-review process)

Tool 4: Packages to create reproducible reports and interactive apps, like R markdown and Shiny. These allow you to:

✓ Report on your data selection rules or data analyses to your research group and therefore increase your reliability
✓ Easily explain code to a teammate with whom you exchange and check codes (a “code buddy”)
✓ Create interactive web pages from your dataset so that collaborators can easily engage with and use this dataset
✓ Combine data cleaning and analysis into a single reproducible document

## CONCLUSION: EMBRACE AND INCENTIVIZE OPEN SCIENCE TO MAXIMIZE OUR CREDIBILITY

The Open Science movement requires a collaborative effort between journals and editors, reviewers, funding agencies and institutions, and us as researchers. Funding agencies and institutions can represent enforcement and facilitation through demanding data and software management plans and fund institutional or regional research software engineers to provide guidance, training and technical support. Journal editors can join the growing number of journals adhering to the Transparency and Openness Promotion (TOP) guidelines developed by the COS (https://cos.io/top/#list, Nosek *et al.* 2015) and embodied for our field by the Tools for Transparency in Ecology and Evolution checklist (TTEE, https://osf.io/g65cb/).

Crucially, however, our primary responsibility as reviewers and authors is to use, teach, and encourage good practices and constantly improve our own scientific methods. The guidelines and framework that we have presented in this paper (Open Science section, Table 1, Box 2) represent a comprehensive toolkit to help researchers take their first (or further) steps towards reliable, reproducible, and replicable science.

The field of behavioral ecology, almost uniquely, expects variation in responses. We need to adopt rigorous methods to be able to tease apart this variation from random noise and to produce credible results.

## FUNDING

This work was supported by the Natural Environment Research Council (MO 005941), the Volkswagen Foundation, the Engineering and Physical Sciences Research Council to M.C., the Software Sustainability Institute to M.C., and by Mozilla Science Lab to A.K.
